# Functional Fruit Snacks Enriched with Natural Sources of Fructooligosaccharides: Composition, Bioactive Compounds, Biological Activity, and Consumer Acceptance

**DOI:** 10.3390/molecules30122507

**Published:** 2025-06-07

**Authors:** Paulina Nowicka, Michalina Marcińczak, Martyna Szydłowska, Aneta Wojdyło

**Affiliations:** Department of Fruit, Vegetables and Plant Nutraceutical Technology, Faculty of Biotechnology and Food Science, Wrocław Univeristy of Environmental and Life Sciences, 37 Chelmonskiego, 51-360 Wrocław, Poland; 116487@student.upwr.edu.pl (M.M.); martyna.szydlowska@upwr.edu.pl (M.S.); aneta.wojdylo@upwr.edu.pl (A.W.)

**Keywords:** fruit leather, Jerusalem artichoke, chicory, FOS, polyphenols, sensory evaluation

## Abstract

This study aimed to develop innovative fruit leather with programmed health-promoting properties, enriched with fructooligosaccharides (FOS) from chicory and Jerusalem artichoke. Their physicochemical properties were assessed, including the profile of polyphenolic compounds, pro-health effects, and sensory characteristics. The products contained various fruits (including pear, red currant, peach, and haskap berry) and 10% FOS powders. It was shown that the addition of FOS reduced acidity and total sugar content while increasing fiber content—especially fructans—and selected minerals (K, Mg, Zn). The addition of FOS also modulated the profile of polyphenolic compounds, whereas fruit leather without FOS was characterized by a higher concentration of these compounds. It was shown that the addition of chicory significantly modulates the ability to inhibit α-glucosidase. At the same time, in the case of the Jerusalem artichoke, the inhibition efficiency depends on the type of fruit matrix. Sensory-wise, the highest scores were given to recipes without FOS additives, with Jerusalem artichoke being better accepted than chicory. The results indicate the potential of using FOS as a functional additive, but their effects on taste and texture require further optimization.

## 1. Introduction

There is an increase in obesity, type 2 diabetes, cardiovascular disease, cancer, and neurodegenerative diseases among the general public. The main causes of these diseases are considered to be a sedentary lifestyle and the consumption of highly processed food. However, the nutritional awareness of consumers is increasing. More and more attention is being paid to the fact that food does not only serve to satisfy hunger. Bioactive ingredients in food products—such as minerals, vitamins, dietary fiber, and natural antioxidants—contribute to maintaining and even improving human health.

Additionally, with the development of food science, the functional food market, and the growing demand from consumers, there is a trend to design and implement food products that are both sensory-attractive and have appropriate nutritional value, rich in active substances with health-promoting properties. An example of such products is fruit leather, which, if well-designed, can serve as a healthy snack for both children and adults.

Fruit leather, also called fruit roll or fruit bar, is defined as a snack made by drying concentrated fruit puree or fruit juice concentrate that is distributed in a thin, even layer. Such snacks can be produced from one or more types of raw materials, with or without added sugar, thickeners, or antioxidants [1,2]. It is also a product that can be freely fortified, and its composition can be modulated—an attractive feature from the perspective of designing innovative food with programmed health-promoting properties. One promising direction for shaping both the sensory characteristics and nutritional values of these products is the application of rich sources of fructooligosaccharides (FOS) to the formulation. This direction has not been previously explored and seems extremely promising for the design of new foods.

FOS are short-chain carbohydrates belonging to the group of fructans—low-molecular-weight polymers of β-fructofuranose. Fructans are composed of D-fructose monomers linked by β-(1→2)-glycosidic bonds. At the terminal end of the chain is a sucrose molecule, the fructose residue of which is linked to the fructofuranose chain by a β-(2→1)-glycosidic bond [3,4]. FOS are known primarily for their prebiotic properties. Like other prebiotics, they are fermented in the large intestine to short-chain fatty acids (SCFAs) by bifidobacteria. The resulting SCFAs lower the pH of the intestinal contents, which inhibits the development of harmful microorganisms such as *Escherichia*, *Clostridium*, *Listeria*, *Salmonella*, or *Shigella*, along with the carcinogenic compounds they may produce. Additionally, SCFAs inhibit the proliferation of cancer cells. Prebiotics also improve intestinal peristalsis, regulate defecation, and increase the fecal mass [3,5].

FOS is also classified as a soluble fraction of dietary fiber. They bind water and create viscous solutions. FOS has been shown to enhance the absorption of elements such as magnesium, iron, calcium, and zinc. Increased calcium absorption has been observed in postmenopausal women, potentially delaying bone demineralization. FOS is also reported to regulate lipid, cholesterol, and glucose metabolism [4,6,7].

Beyond their health-promoting effects, FOS possess technological properties that make them useful in the food industry. They are highly water-soluble and can bind moisture. Products containing FOS tend to have a longer shelf life and reduced microbial growth. Moreover, FOS are stable in food products at a pH range of 4–7 and temperatures up to 140 °C. Their technological properties are similar to glucose syrup, and they are more viscous than sucrose solutions of the same concentration due to their higher molecular weight. FOS has a sweetness of 0.3–0.6 times that of sucrose, with a mild sweet taste, making them suitable for low-calorie, reduced-sugar products [3,8].

The richest sources of FOS include Jerusalem artichoke and chicory. Chicory (*Cichorium intybus* L.) is a perennial plant from the *Asteraceae* family. About 75% of the chicory root mass is water, and carbohydrates make up 80–90% of the dry mass. Inulin, the main reserve sugar of chicory, accounts for about 20% of the fresh root mass. The dietary fiber content is about 5% of the root mass, and the protein content is about 4%. Chicory is a source of sesquiterpene lactones—bitter-tasting compounds with documented health benefits. The root contains phenolic compounds (20 mg of gallic acid/g of extract), mainly caffeic acid, chlorogenic acid, and coumaric acid derivatives. It also contains minerals such as calcium (181.26 mg/100 g), potassium (103.70 mg/100 g), magnesium (20.14 mg/100 g), and trace elements including sodium, iron, copper, and zinc. Chicory also contains volatile organic compounds, with kaempferol, octane, and p-cymene among the most prominent [9,10,11]. Jerusalem artichoke (*Helianthus tuberosus* L.), also a member of the Asteraceae family, is a perennial whose tubers are composed of 75–80% water. Carbohydrates constitute 15–17% of the mass, with nearly 80% of that being inulin. Jerusalem artichoke contains about 2% protein and all essential amino acids. The tubers contain significant amounts of potassium (23,356 mg/kg dry weight), phosphorus (3892 mg/kg dry weight), calcium (1864 mg/kg dry weight), iron, and silicon [12]. Jerusalem artichoke contains β-carotene, vitamin C and thiamine, riboflavin, niacin, and biotin. The nutritional value of Jerusalem artichoke is 73 kcal/100 g.

According to the above, the use of rich sources of FOS (such as powdered chicory and Jerusalem artichoke) is a promising way to enrich functional foods. Until now, similar solutions have not been used in food technology, but they may have a positive impact on both the efficiency of the process and the quality of final products. The application of the rich FOS sources in the recipe will enable the modification of the product’s physical and health-promoting properties, giving them new, unique characteristics in line with market and current consumer needs. The creation of fruit leathers in addition to FOS is also a market reaction to the growing demand for high-fiber, low-sugar, and prebiotic foods, which is very important for appropriate nutrition programs for children and adolescents, especially those with obesity, diabetes or problems with defecation. Hence, the present research aimed to design innovative and functional fruit leathers incorporating rich sources of FOS—namely, chicory and Jerusalem artichoke. It was hypothesized that adding these FOS-rich ingredients could modulate the consistency and quality of fruit leather. Furthermore, the health-promoting effects of FOS may influence the nutritional and dietary value of the final products, including fiber content, bioactive compound profile, antidiabetic potential, and antioxidant activity.

## 2. Results and Discussion

### 2.1. Physicochemical Properties of Obtained Products

Table 1 presents selected physicochemical parameters of the obtained fruit leathers, including color, dry mass, water activity, energy value, titratable acidity, and pH.

A significant parameter influencing the sensory desirability of the product is its color. The prepared fruit leather variants retained the characteristic color of the starting raw materials. Among the tested samples, the highest lightness (L* value) was observed in peach fruit leather without added FOS (52.16), while fruit leathers made from haskap berries had the darkest appearance. No statistically significant difference was observed between the FOS-containing and FOS-free haskap berry recipes. In contrast, the addition of FOS increased the darkness (i.e., reduced L* value) in red currant and peach fruit leathers. Plum fruit leathers became lighter upon the addition of chicory and Jerusalem artichoke powders.

The a* parameter determines the intensity of the red color. The highest value of the parameter was read for red currant fruit leathers without added FOS (43.66). The highest share of green (lowest a* value) was noted in haskap berry fruit leathers without FOS (1.26). For the red currant, peach, and plum variants, the addition of chicory and Jerusalem artichoke powders increased the green component. The b* parameter measures the intensity of yellow versus blue. Peach fruit leathers without added FOS were characterized by the highest value of this parameter, amounting to 49.10. The highest share of blue color was observed for haskap berry fruit leathers (0.30 to (−0.07)). No statistical difference was found between the recipe variants of the haskap berry leathers. On the other hand, it was observed that the addition of FOS increased the share of blue color in the leathers of red currant, peach, and plum.

This study also evaluated the dry matter content of the final formulations, which ranged from 87.30% (peach with chicory) to 89.91% (red currant with Jerusalem artichoke). According to Nowicka et al. [13], dried fruit should contain 20–25% water to inhibit microbial growth. In practice, even a lower moisture content is preferred to limit nonenzymatic browning. The fruit leathers obtained in this study had a moisture content ranging from 10.09 to 12.71%, well below typical dried fruit standards, indicating a high level of dehydration and better microbial stability. This was supported by low water activity values, which ranged from 0.309 (red currant with chicory) to 0.333 (plum with Jerusalem artichoke). No statistically significant differences in water activity were observed between the tested variants.

For comparison, early studies of papaya fruit leathers reported water activity between 0.500 and 0.520 [14]. Quintero Ruiz et al. [15] found apple leathers had a water activity of approximately 0.700 (2016) reported values from 0.460 to 0.542 in plum–chokeberry fruit leathers with added black currant or raspberry, and water activity ranged from 0.460 to 0.542. Gonzales-Herrera et al. [16] studied the water activity of apple leathers with the addition of agave fructans, inulin, and oligofructose. The addition of inulin and oligofructose resulted in lower water activity (0.478), while the water activity of products with the addition of agave fructans was higher (0.696). The water activity determined in this study was therefore similar to most previous studies of fruit leathers. Both literature and experimental values of water activity classify fruit leathers as low-water foods. The above allows to conclude that the developed fruit leathers will be microbiologically and enzymatically stable for at least 6 months, which is directly due to the water activity in these formulations being below 0.6 [17].

The energy values of the obtained snacks ranged from 327.84 to 338.96 kcal/100 g. No statistically significant differences were observed between the variants with and without FOS addition.

Acidity (Table 1) and sugar content (Table 2) were also analyzed in the fruit leathers. Acidity is an important physicochemical parameter that, along with sugar content, shapes the overall taste of fruit products. The highest total acidity was recorded in red currant fruit leathers (11.12 g malic acid/100 g). It was observed that the addition of FOS led to a decrease in the acidity of all fruit leather variants compared to the corresponding products without FOS, indicating that the additive modulated this parameter in the final product. The lowest total acidity was found in peach leathers enriched with Jerusalem artichoke powder (3.28 g malic acid/100 g).

The enrichment of fruit leather recipes with FOS involved replacing 10% of the mixture with chicory or Jerusalem artichoke powders, which effectively reduced the share of fruit purees—naturally rich in organic acids—by the same amount. This reduction contributed to the observed decrease in total acidity in the FOS-containing recipes.

In contrast, the highest total sugar content was found in haskap berry leathers without FOS (23.44 g/100 g), while the lowest was observed in plum leathers with Jerusalem artichoke powder (13.55 g/100 g). Fructose was the dominant sugar across all developed variants. The recipes also contained glucose (0.66–2.39 g/100 g) and small amounts of sucrose (0.02–0.72 g/100 g). Sorbitol was also detected in small quantities, with the highest levels observed in plum-based variants (0.90–0.71 g/100 g). In haskap berry leathers without FOS, galactose was identified at 0.09 g/100 g. Small amounts of mannose were found in red currant, plum, and peach leathers after enrichment with chicory powder.

It was observed that the addition of FOS to fruit leathers resulted in a decrease in total sugar content. This effect is likely due to the partial replacement (10%) of fruit puree with FOS-rich powders. Notably, the content of fructose and sorbitol decreased. The addition of chicory powder increased the share of glucose in each of the tested recipes. It was noted that the addition of Jerusalem artichoke powder increased sucrose content. Enriching the recipes with FOS resulted in a decrease in the fructose content in the tested products. A similar relationship is described by Sánchez Riaño et al. [18]. The authors indicated that the content of reducing sugars in mango fruit leathers was several times higher than in the pulp of fresh fruit. The addition of complex sugar polymers with hydrocolloid properties (including maltodextrin, gum arabic, and carboxymethylcellulose) resulted in a decrease in the content of reducing sugars compared to mango fruit leathers without additives. The content and diversity of sugars present in the developed fruit leather recipes result exclusively from the content of sugars naturally occurring in the raw materials used. The factors influencing the sugar content in fruit are varietal characteristics, degree of ripeness, and time of harvest. During the process of thickening and drying, the fruit mass undergoes dehydration, which is associated with the simultaneous concentration of minerals and nutrients. During heating, acid hydrolysis of sucrose to fructose and glucose can also occur. Therefore, the total sugar content in fruit leathers is higher than that determined in fresh fruit. Unlike conventional sweet snacks rich in sucrose and sugar syrups, the dominant sugar in each variant of prepared fruit leathers was fructose. The presence of easily digestible simple sugars means that fruit leathers meet the criterion of a snack as a product providing energy between meals. Replacing 10% of the fruit puree mass with the addition of FOS contributed to the creation of fruit leathers with reduced sugar content. Products with these characteristics are desired by consumers who pay attention to the nutritional value of the food products they consume.

### 2.2. Content of Dietary Fiber in Fruit Leathers

Table 2 presents the results of dietary fiber content in the obtained products, including both insoluble and soluble fractions—specifically pectin and fructans.

The content of the insoluble fiber fraction was primarily determined by the fruit matrix rather than the addition of FOS. Accordingly, the products with the highest concentration of this component were leathers based on haskap berry >> red currant >> plum >> peach. This trend accurately reflects the natural distribution of insoluble fiber in the raw fruits used, which were the main ingredients in the final products [19,20].

A similar trend was observed for pectin content, representing the soluble fiber fraction. The highest pectin content was found in peach and red currant fruit leathers without FOS (10.68% and 10.43%, respectively). In contrast, the lowest pectin levels were recorded in haskap berry fruit leathers with added chicory powder (5.28%). The addition of FOS was associated with reduced pectin content, with chicory powder leading to a greater reduction than Jerusalem artichoke powder. Fruits are naturally rich in pectins, and among the raw materials used, red currant, peach, and plum contain the highest amounts. Replacing 10% of the fruit puree with FOS led to a proportional decrease in pectin content, due to the lower amount of fruit-based material in the mixture.

In contrast, a completely different pattern was noted for fructan content—another key soluble fiber fraction. The concentration of this fraction was mainly influenced by the addition of Jerusalem artichoke and chicory. Leathers enriched with Jerusalem artichoke had a significantly higher fructan content than those with chicory. Trace levels of fructans were found in fruits like peach and haskap berry, while they were undetectable in plum and red currant.

These findings confirm that the incorporation of FOS-rich ingredients can significantly shape the final fiber composition of fruit leathers, especially in increasing the proportion of fructans, which are recognized for their prebiotic properties. Ultimately, the most fiber-rich formulations, including high fructan content, were those based on berries (haskap berry and red currant) enriched with Jerusalem artichoke, containing 31.22% and 28.64% dietary fiber, respectively.

Although the addition of a rich source of FOS did not significantly reduce the product’s energy value, there have been significant qualitative and quantitative changes in reduced sugars, and dietary fiber. The enriched fruit leathers were characterized by significantly lower sugar concentrations and increasing fiber content at the same time, focusing on soluble components such as fructans. The addition of chicory and Jerusalem artichokes to fruit leathers changed chemical properties, resulting in high dietary fiber content, reduced sugar content, and significant positive changes in prebiotic component modulation. In total, this allowed for the development of functional snack products that have a potentially beneficial impact on the human body in terms of the prevention of obesity, diabetes, and digestive diseases. Consuming products rich in dietary fibers, especially those containing their soluble fraction, has many health benefits. Firstly, fiber helps to regulate the functioning of the intestine. In addition, the prebiotic properties are widely recognized, modulating the development of intestinal microflora, which has a direct positive effect on the functioning of the digestive tract and strengthening human immunity. In addition, soluble fibers have beneficial effects on carbohydrate metabolism, reducing glucose absorption and maintaining a stable blood sugar level [4,5,6]. This formulation has not been designed to date, but it can actually program good dietary habits for different groups of consumers.

### 2.3. Mineral Content in Obtained Products

The content of selected mineral components in fruit leathers, with or without the addition of fructooligosaccharides (FOS), is presented in Table 3.

The potassium content in fruit leathers increased after enriching the recipes with FOS. The highest amount of this element was determined in red currant fruit leathers with the addition of Jerusalem artichoke powder (2874.02 mg/100 g). The lowest content of the discussed element was found in the fruit leathers of haskap berries and plums without additives, amounting to 1360.09 and 1291.56 mg/100 g, respectively. Chicory root contains 103.70 mg K/100 g [21], and Jerusalem artichoke tuber contains 429.00 mg K in 100 g of fresh mass [22], hence the addition of Jerusalem artichoke significantly modulated the final value of this macroelement. In turn, the addition of chicory powder also led to an increase in sodium content in fruit leathers. The highest sodium content was recorded in red currant leathers enriched with chicory (12.18 mg/100 g), while the lowest was found in plum leathers without FOS (2.17 mg/100 g).

It was also observed that the addition of FOS increased the magnesium content in fruit leathers, with the highest values being characteristic of recipes containing chicory powder. The highest amounts of the discussed element were determined in peach leathers with the addition of chicory powder (310.96 mg/100 g), and the lowest in the fruit leathers of haskap berries without additives (106.25 mg/100 g).

Chicory powder also enhanced calcium content in red currant, peach, and plum leathers. However, the addition of Jerusalem artichoke powder resulted in decreased calcium content in haskap berry, red currant, and peach leathers compared to their FOS-free counterparts. The highest calcium concentration was found in red currant leather with chicory (44.96 mg/100 g), while the lowest was observed in plum leather without additives (11.77 mg/100 g).

The highest iron content was detected in red currant leathers without additives and with Jerusalem artichoke powder (6.34 and 6.27 mg/100 g, respectively), and the lowest in plum leathers (5.28 mg/100 g).

Overall, FOS addition led to an increase in zinc content across all recipes, with higher values in products containing chicory powder. The highest zinc content was found in peach leather with chicory powder (0.91 mg/100 g), and the lowest in plum leather without FOS (0.23 mg/100 g). The addition of chicory increased the manganese content in fruit leathers regardless of the recipe, while the addition of Jerusalem artichoke resulted in a decrease in the content of this microelement in fruit leathers from haskap berries, peaches, and plums. The richest in manganese were fruit leathers from haskap berries and red currants enriched with chicory powder (0.61 mg/100 g). The lowest content of the discussed element was determined in peach leathers with the addition of Jerusalem artichoke powder (0.26 mg/100 g).

The content of mineral components depends on a variety of factors and the conditions of growing the raw material. Changes in the content of mineral components in fruit leathers with the addition of FOS compared to recipes without additives may be caused by a decrease in the content of fruit purees in the mixture. Technological processes accompanying the processing of fruit to obtain fruit leathers affect the concentration of mineral components due to the removal of significant amounts of water. Regardless of the recipe, the resulting fruit leathers were characterized by high potassium levels (1291.56–2874.02 mg/100 g). Both chicory and Jerusalem artichoke powder contributed to increased potassium content. Notably, chicory powder consistently enhanced sodium, magnesium, zinc, and manganese levels compared to both Jerusalem artichoke-enriched and control samples.

### 2.4. Content of Polyphenols in Obtained Products

**Figure 1:**
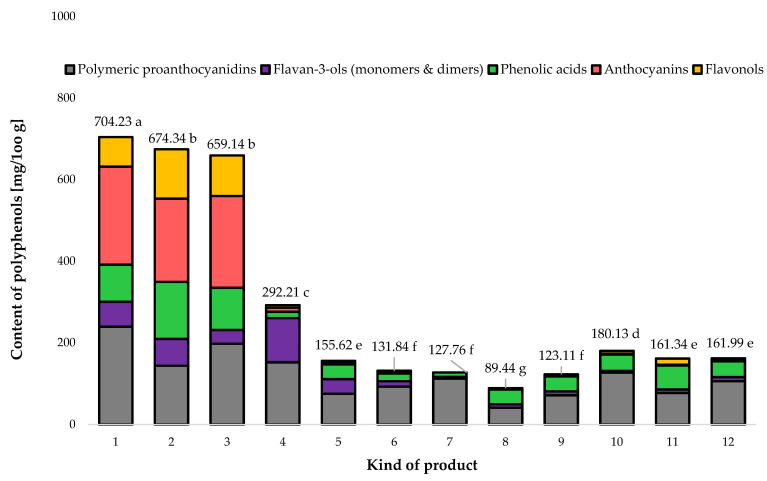
Content of polyphenols in obtained fruit leathers. Data are given as mean (*n* = 3). Mean values above the chart with different letters (a–g) are significantly different (homogenous groups) at *p* ≤ 0.05.

Figure 1 and Table S1 present the content of polyphenolic compounds in the developed fruit leathers, including the following fractions: anthocyanins, flavonols, phenolic acids, flavan-3-ols, and procyanidin polymers.

Among all tested variants, haskap berry leathers showed the highest anthocyanin content (240.16–203.98 mg/100 g). Small quantities of anthocyanins were found in red currant leathers (10.03 mg/100 g) and plum leathers (1.93–2.36 mg/100 g). In recipes containing FOS, the anthocyanin content decreased in haskap berry and red currant leathers, while no statistically significant change was observed in plum-based leathers. These findings suggest that anthocyanin concentration in the final product is primarily determined by the type and proportion of fruit used.

In fresh haskap berries of the ‘Aurora’ variety, anthocyanin content was previously reported as 112.37 mg/100 g [23]. Djordjević et al. [24] indicated that the content of anthocyanins in red currant fruits of the ‘Random’ variety is 12.40 mg/100 g of fresh weight. Other raw materials included in the discussed products are characterized by low concentrations of anthocyanins.

The highest amount of flavonols was found in haskap berry fruit leathers (121.03–72.53 mg/100 g). Flavonols were not detected in peach fruit leathers without additives, whereas small amounts were present in variants containing chicory and Jerusalem artichoke powders (3.54 and 5.07 mg/100 g, respectively). In this case, fortification of the final formulations was achieved through the addition of FOS-rich ingredients.

Both Jerusalem artichoke and chicory also increased the concentration of phenolic acids in the developed fruit leathers, with chicory powder producing a greater increase than Jerusalem artichoke. The highest phenolic acid content (139.71 mg/100 g) was observed in haskap berry leathers with chicory. The lowest content was recorded in peach leathers without additives (10.97 mg/100 g). Massoud et al. [21] reported that in dry chicory root extract (20 mg GAE/g), nearly 28% and 25% were m-coumaric and p-coumaric acids, 25% caffeic acid, and about 11% chlorogenic acid. Showkat et al. [25] found that the phenolic acid content in ethanolic extracts of Jerusalem artichoke tubers ranged from 0.9 to 1.4 mg GAE/g dry weight.

The addition of chicory and Jerusalem artichoke also reduced the final concentration of polymerized compounds, while simultaneously increasing the levels of flavan-3-ol monomers and dimers, particularly in formulations with chicory. This effect may result from the breakdown of procyanidin polymers into smaller units. Chicory contains enzymes capable of modifying the structure of complex polyphenolic compounds. Additionally, the high temperatures used during the drying process may lead to the degradation of polymers into simpler forms such as catechin, epicatechin, and procyanidins B1 and B2. Acidic conditions and the presence of antioxidants may further facilitate this transformation.

It is also important to note that interactions between polyphenolic compounds from different fractions often result in a dynamic equilibrium between oligomers and monomers of procyanidins [26,27].

In general, it was shown that while the addition of FOS-rich sources (chicory and Jerusalem artichoke root) can modestly influence the distribution of polyphenolic fractions, the main contributor of these bioactive compounds remains the fruit component of the recipe.

### 2.5. Pro-Health Properties of Obtained Fruit Leather

#### 2.5.1. Antioxidant Activity

The antioxidant activity of the tested fruit leathers varied depending on the recipe composition and the content of antioxidant compounds in each fruit variety (Table 4).

The highest antioxidant potential was measured using the ORAC method, while the lowest values were obtained via the FRAP method. Unlike other methods, the values obtained using the ORAC method are the closest to the antioxidant potential of the human body, because it uses the peroxide radical that is dominant in the human body [28]. Regardless of the method used, haskap berry fruit leathers demonstrated the highest antioxidant activity (ranging from 18.76 to 19.56 mmol Trolox/100 g), while peach-based leathers exhibited the lowest (ranging from 2.13 to 3.40 mmol Trolox/100 g). This pattern corresponds directly to the polyphenol content, which significantly influences the final antioxidant potential. A positive correlation was observed between the content of individual fractions of polyphenolic compounds and the oxidation capacity of the ABTS^•+^ cation radical. The highest correlation was shown by flavan-3-ols, while the content of procyanidin polymers was not positively correlated with the increase in antioxidant potential determined by this method. The same correlation between the content of polyphenolic compounds and antioxidant activity was observed in the case of the FRAP method. A positive correlation was observed between the content of anthocyanins, flavonols, phenolic acids, and flavan-3-ols and the antioxidant activity tested by the ORAC method. Therefore, an increase in the content of polyphenolic compounds translates into a stronger antioxidant potential. The addition of FOS-rich ingredients reduced the antioxidant activity of fruit leathers as determined by the ABTS and FRAP methods. However, in the ORAC assay, chicory powder enhanced the antioxidant activity of red currant leathers, and Jerusalem artichoke powder increased the antioxidant activity in haskap berry leathers. Dalar and Konczak [29] reported that the water-alcohol extract of chicory root exhibits an antioxidant capacity of approximately 900.00 μmol Trolox/g dry weight. The antioxidant activity of Jerusalem artichoke tuber, as determined by the FRAP method, is 17.39 mg Trolox/g fresh weight, with above-ground parts showing even higher antioxidant potential [30].

The reduction in antioxidant activity in FOS-enriched leathers is likely due to the decreased proportion of fruit purees in these formulations. Fruit leathers with added FOS showed lower levels of polyphenolic compounds, which are strong antioxidants. Furthermore, the antioxidant activity of chicory root and Jerusalem artichoke tuber is not significant.

#### 2.5.2. Ability to Inhibit α-Amylase and α-Glucosidase

This study also assessed the ability of the developed fruit leathers to inhibit α-amylase and α-glucosidase—key enzymes involved in regulating postprandial blood glucose levels. The results are presented as IC_50_ values (mg/mL) in Table 4.

Analysis of the data showed that the formulations were generally more effective inhibitors of α-glucosidase than α-amylase. The strongest inhibitory effect on α-glucosidase activity was observed in products containing chicory as a rich source of FOS. Notably, formulations combining chicory with haskap berry, red currant, and peach (products 2, 5, and 8) achieved IC_50_ values below 0.5 mg/mL, indicating very high inhibitory potential—approaching that of acarbose, a widely used pharmaceutical inhibitor of these enzymes in the treatment of diabetes. The observed effect is likely due to the direct action of polyphenolic compounds on α-glucosidase. Correlation analysis revealed a positive relationship between the inhibitory effect on this enzyme and all polyphenolic fractions, with the strongest correlation observed for anthocyanins and flavan-3-ols (R = 0.450), followed by flavonols (R = 0.420), and polymeric proanthocyanidins and phenolic acids (R > 0.300). However, it should be noted that individual compounds with specific structures, rather than the entire complex of compounds, are probably responsible for the greatest inhibitory effect on α-glucosidase. Although the correlation coefficient for phenolic acids was R = 0.324, chlorogenic acid, the primary phenolic acid in chicory, showed a much higher correlation of R = 0.972. This indicates a very strong potential of chlorogenic acid for direct inhibition of α-glucosidase. The inhibitory mechanism of chlorogenic acid is well documented and involves hydrophobic and π–π interactions with aromatic residues of the enzyme, as well as hydrogen bonding between the hydroxyl and carboxyl groups of chlorogenic acid and amino acid residues within the enzyme’s active site [31,32]. These interactions lead to the formation of a stable enzyme-inhibitor complex that restricts substrate access to the active site. Additionally, other researchers suggest that in the case of chicory root, the inhibitory effect may be further enhanced by lactone sesquiterpenes, which contribute to the characteristic bitterness of chicory [29,33].

It is worth noting that the fruit leathers without FOS additives exhibited significantly higher IC_50_ values and thus lower inhibitory activity. Among these, berry-based formulations (red currant and haskap berry) were more effective inhibitors of both α-amylase and α-glucosidase compared to stone fruit-based products.

However, in the case of Jerusalem artichoke, varied efficacy against enzymes was observed, depending on the type of fruit used as a base. The observed variation in the efficacy of Jerusalem artichoke as an inhibitor of α-amylase and α-glucosidase enzymes, depending on the type of fruit matrix used, may result from several biological and chemical factors. First of all, the possibility of interactions between bioactive components contained in both Jerusalem artichoke, including FOS, and in the fruit itself should be taken into account [34]. These compounds can mutually influence each other’s biological activity and, in some cases, form complexes that change the efficiency of enzyme inhibition. The pH of the environment may also play an important role, as it differs between fruits and can affect the stability of enzymes and their natural inhibitors [35]. In addition, fruits contain different amounts of soluble and insoluble fiber, which, in combination with inulin present in Jerusalem artichoke, can modify the physicochemical properties of the matrix, such as viscosity or the availability of enzyme substrates [36].

Together, these factors likely explain the observed differences in IC_50_ values and confirm that the effectiveness of functional additives such as Jerusalem artichoke is strongly dependent on the physicochemical and biochemical characteristics of the fruit matrix used [34].

##### Sensory Evaluation of Obtained Fruit Leathers

**Figure 2:**
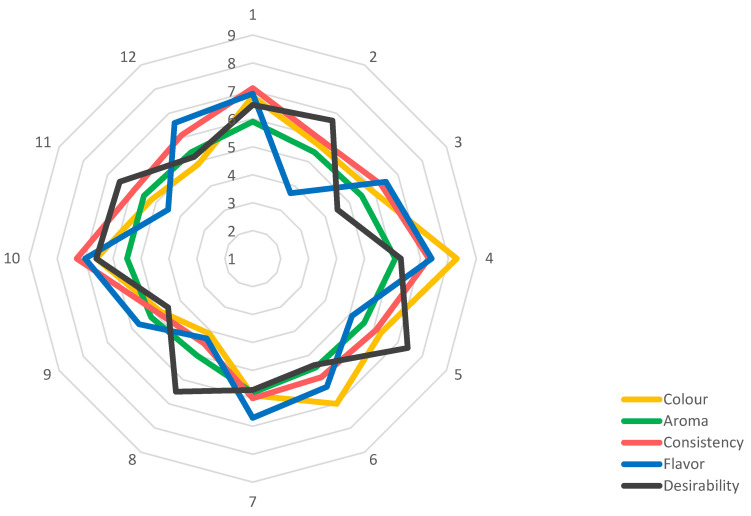
Sensory evaluation of obtained fruit leather.

Figure 2 presents the results of the sensory evaluation of the prepared fruit leather variants based on key quality attributes—color, smell, consistency, taste, and overall assessment.

Red currant fruit leather without additives received the highest acceptability score for color (8.3). A similarly high rating was given to the red currant variant enriched with Jerusalem artichoke powder (7.0), falling within the “I like” range. In contrast, the lowest color score was assigned to peach fruit leather with added chicory powder (4.1). The addition of FOS influenced color perception to vary degrees, a trend also observed in the objective color analysis using the CIE L* a* b* method.

The aroma of the fruit leathers, regardless of the variant, fell within the range between “I neither like nor dislike” and “I like a little” (5.0–6.0). Peach leathers with Jerusalem artichoke powder received the lowest aroma rating (5.2), while red currant leathers without additives were the only variant to score slightly above average (6.1). Overall, the smell of the snacks was not intense, and the fruit odor was not noticeable, so the assessment of this quality parameter was neutral. The direct cause of this phenomenon lies in the way the process was carried out. Obtaining fruit leather involves an initial thickening of the purees, followed by a drying stage. The combination of high temperature and the presence of oxygen leads to the loss or oxidation of volatile aromatic compounds, which directly contributes to a decline in aroma quality. In the future, it may be worth considering the use of combined drying techniques—such as microwave-vacuum drying or freeze-drying—to better preserve volatile compounds and prevent their degradation. However, this would significantly increase the overall processing costs.

According to the assessors, the most acceptable consistency was observed in red currant and plum fruit leathers without the addition of FOS (score: 7.3). Enriching the recipes with FOS led to a decrease in product acceptability. Among the FOS additives, Jerusalem artichoke powder was more acceptable than chicory powder. The lowest consistency scores were assigned to fruit leathers enriched with chicory powder, regardless of the fruit used. Peach leather with added chicory received the lowest score (4.5). González-Herrera et al. [16] and García-García et al. [37] studied the effect of inulin, agave fructans, and oligofructose on the properties of apple fruit leathers. Their findings, based on both sensory evaluation and mechanical measurements, revealed that inulin-enriched apple leathers had higher hardness compared to those containing agave fructans and oligofructose. Snacks with agave fructans showed more desirable consistency and were better accepted by consumers than those with inulin. Therefore, the addition of chicory powder, which contains inulin, affects the change in the properties of quality parameters, which translates into a decrease in the acceptability of snacks by consumers.

Red currant, plum, and haskap fruit leathers without FOS were rated highest for taste, receiving scores of 7.4, 7.0, and 6.9, respectively. The addition of FOS generally reduced consumer acceptability in terms of taste. Across all recipe variants, fruit leathers enriched with chicory powder scored lower than those with Jerusalem artichoke powder. The lowest taste score (3.7) was given to haskap berry fruit leathers containing chicory. The distinctive, bitter taste of chicory is undesirable in fruit-based snacks and likely contributed to the lower sensory ratings of these variants.

Pereira et al. [38], in developing a skyr yogurt recipe with mango puree, natural sweeteners, and FOS, reported no significant effect of FOS on consistency and taste. However, consumer preference was higher for skyr with FOS sweetened with thaumatin, highlighting the influence of sweetener type on product acceptability. Therefore, the addition of FOS may modulate the sensory properties of products that are rich in them. The presence of other ingredients that determine taste sensations is also important in the reception of products by potential consumers. In the future, to reduce bitterness in the developed formulations, it may be worth considering the use of various pre-treatment techniques or the addition of flavor maskers. For pre-treatment, a brief blanching of chicory appears to be a reasonable approach, as it may help reduce bitterness. Additionally, removing the core of the chicory—where the bitter taste is most concentrated—could also be beneficial. The use of natural flavor maskers such as spices, herbs, or sweeteners is another option worth exploring.

In the overall assessment, the highest rating was given to red currant fruit leathers without added FOS (7.4). The lowest ratings were assigned to haskap berry and peach leathers enriched with chicory powder (4.5). It was observed that each flavor variant of fruit leather without additives received higher scores compared to its FOS-enriched counterpart. Among the FOS types, formulations with Jerusalem artichoke powder were more acceptable to consumers than those with chicory powder. Based on these findings, it can be concluded that fruit leather recipes without FOS were rated highest across all quality characteristics. Recipes enriched with Jerusalem artichoke powder were generally better accepted than those containing chicory powder.

Fruit leathers are an innovative snack that is gaining popularity. The addition of FOS to the developed recipe variants modulated the physicochemical parameters, health-promoting properties, and quality characteristics of the products. Fruit snacks enriched with FOS known for their prebiotic effects may increase consumer interest in this product, but consumer acceptability of the final product is also important. The conducted studies show that Jerusalem artichoke powder had the greatest potential in creating commercial fruit leathers.

## 3. Materials and Methods

### 3.1. Chemicals and Reagents

Acetonitrile, methanol, ethanol, and isopropanol used for HPLC analysis, as well as standards of sugars and organic acids, were purchased from Merck (Darmstadt, Germany). Reagents used for the determination of health-promoting properties via in vitro assays—including acarbose, α-amylase from porcine pancreas (type VI-8), p-nitrophenyl-α-D-glucopyranoside, α-glucosidase from *Saccharomyces cerevisiae* (type I), DMSO, ascorbic acid, phloroglucinol, TPTZ, AAPH, fluorescein disodium, Trolox, potassium persulfate, disodium and dipotassium phosphate, and potato starch—were purchased from Sigma-Aldrich (Steinheim, Germany). Standards of phenolic compounds used for the determination and identification of bioactive compounds in fruit leather were obtained from Extrasynthese (Genay, France) and TRANS MIT GmbH (Giessen, Germany). Acetone, oxalic acid, and formic acid were purchased from CHEMPUR (Piekary Śląskie, Poland).

### 3.2. Sample Preparation

**Scheme 1:**
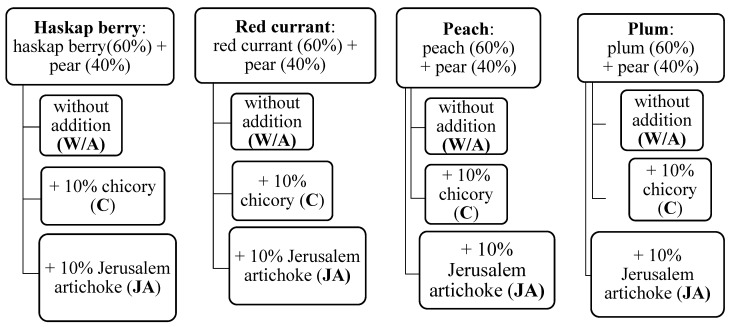
Fruit leather variants with and without the addition of rich sources of FOS.

The research material consisted of fruit leathers prepared by air drying fruit puree mixtures with or without the addition of FOS (see Scheme 1 for the list of products), fruit puree mixtures (peach, pear, plum) were prepared using raw materials obtained from the branch of the Central Research Center for Cultivar Research in Zybiszów near Wrocław (51°04′ N, 16°78′ E). Harvesting was conducted in the second half of July and the second half of August 2024. Haskap berries (early July 2024) and red currants (August 2024) were purchased from a plantation located near Trzebnica (51°19′ N, 17°03′ E). The characteristics of the plant raw materials used in this study are presented in Tables S2 and S3, which are attached as a supplement to this manuscript.

The following raw material varieties were used:

- ‘Faworytka’ cv. pear,
- ‘Aurora’ cv. haskap berry,
- ‘Random’ cv. red currant,
- ‘Harbinger’ cv. peach,
- ‘SL 3 (Polinka)’ cv. plum.

The selection of individual raw materials resulted directly from the assumptions of this study. The main aim of this work was to obtain snack products, with a high content of dietary fiber, which will be attractive to a wide group of consumers, but especially to children and adolescents. For this reason, pear was chosen as the base. Pear is a sweet raw material that accepts other flavors very well—it is an excellent base for products, emphasizing their taste and providing a balanced background. The remaining raw materials constitute two groups—stone fruits (peach and plum) and berries (haskap berry and red currant) with documented composition in the context of dietary fiber content (its high content).

As a source of FOS, powders previously prepared from chicory and Jerusalem artichoke were added to the recipe. These raw materials were freeze-dried and then crushed using analytical mills. They were purchased from retail sources. The production of fruit leather with the addition of FOS was conducted in three main stages.

#### 3.2.1. Preparation of Fruit Purees

The obtained raw materials were washed, and inedible parts such as seed nests and pits were removed. The raw materials were ground using a Thermomix TM6 (Vorwerk, Wuppertal, Germany) for 30 s at speed 10. To inhibit enzymatic browning, 1% of a 10% ascorbic acid solution was added to the ground fruits. The mixture was then heated to 55 °C at speed 3–4. Afterward, the ground fruits were rubbed through a sieve to obtain a puree.

#### 3.2.2. Preparation of Mixtures

Four flavor variants of fruit mixtures were developed. The base of each recipe was pear puree (40%), supplemented with selected berry and stone fruit purees (Scheme 1). The appropriate purees were weighed and transferred into the Thermomix, then heated for 30 min at 110 °C at speed 4 to achieve thickening. Rich sources of FOS—powdered chicory and Jerusalem artichoke—were added to the cooled purees at 10% of the total mixture mass. The base without any additives served as the control. A total of 12 fruit leather variants were prepared (Scheme 1).

#### 3.2.3. Drying

Each 200 g mixture was spread evenly in a thin layer (3 mm ± 5% thickness) on two trays lined with silicone pads (Wartmann, Tilburg, The Netherlands). Drying was carried out in a Zyle Professional Maxi food dryer (Zyle, Kaunas, Lithuania) at 60 ± 1 °C (air velocity was 1 m/s; relative humidity was 46% ± 2%). The process continued until the leathers were completely dry, with no visible shine and no noticeable stickiness on the surface. After cooling, the leathers (12 types; see Scheme 1) were cut into smaller pieces and stored in airtight containers in a dark, dry place at 20 °C until analysis.

### 3.3. Physicochemical Parameters—Water Activity, Color, Energy Value, pH, Dry Matter Content

The water activity of the obtained products was measured in triplicate using a Novasina water activity meter (LabMas-terav., Lachen, Switzerland) at 20.0 ± 1.0 °C. Dry matter analysis was conducted by the PN-EN 12145:2001 standard [39]. Total acidity was measured via potentiometric titration according to the PN-EN 12147:2000 standard [40]. The pH value and volume of NaOH used were recorded using the TitroLine 6000 titrator (SI Analytics, Mainz, Germany). Total acidity was expressed as the content of malic acid [g]/100 g of the product. All measurements were performed in replicates and are presented as mean ± SD.

The color of the 12 fruit leathers was determined using an A5 Chroma-Meter (Minolta CR300; Osaka, Japan), based on the CIE L* a* b* color space. Lightness (L*), redness-greenness (a*), and yellowness-blueness (b*) values were measured using Illuminant D65 and a 10° standard observer angle. Due to the nonuniform color of the samples, values represent the mean of ten replicates.

The energy value analysis was performed using a C200 calorimeter paired with an RC 2 basic recirculation cooler (IKA, Wilmington, NC, USA). A 1 g sample was placed in quartz crucibles and transferred to a calorimetric bomb, which was sealed and filled with compressed oxygen (~35 bar). The samples were combusted in the calorimeter. If needed, auxiliary materials such as combustion bags, parafilm, or paraffin were used to facilitate combustion. Energy values are reported as the mean of three repetitions.

### 3.4. Determination of Dietary Fiber and Sugar Content

The total dietary fiber (TDF) content in the developed products was determined according to AOAC 991.43 and AACC 32-07 methods using a Fibertec E system analyzer (Fibertec™ 1023, FOSS Analytical, Hillerød, Denmark). Dried and ground samples (1.000 g ± 0.005 g) were weighed in duplicate and covered with 40 mL of MES-TRIS buffer (pH = 8.2). The whole was mixed thoroughly, 50 µL of amylase was added, and the samples were placed in a water bath (100 °C) for 30 min. After this time, the sample was cooled to 60 °C and the second digestive enzyme—protease (100 µL) was added. The material was again placed in a water bath (temp. 80 °C; 30 min) with gentle shaking. Next, 5 mL of 0.561 N HCl was added, and the pH was adjusted to 4.0–4.7. Then, 200 µL of amyloglucosidase was added, and the mixture was incubated at 80 °C for another 30 min. Afterward, 225 mL of ethyl alcohol was added, and the samples were left for 1 h. The material was then filtered using sintered crucibles containing 0.500 g of Celite. The residue retained on the crucible was dried at 108 °C and weighed. The final TDF content was calculated using the following formula:

(1) $$\mathrm{TDF}\;(g/100\;g)=\frac{\frac{R1+R2}{2}-B-P-Ś}{\frac{M1+M2}{2}\times 100}$$

*Ś*—Protein (mg) in the blank

*B*—Protein (mg) in the sample residue

*P*—Ash (mg) in the sample residue

*R*_1_/*R*_2_—Mass of residue on the crucible after drying (mg)

*M*_1_/*M*_2_—Mass of samples (mg) for two replicates.

Pectin content was determined following the procedure described by Pijanowski et al. [41]. Fructans content was determined according to Jaime et al. [42] with slight modifications. A total of 2 g of freeze-dried and milled sample was mixed with 20 mL of 80% ethanol and immediately heated at 100 °C for 10 min. The mixture was centrifuged (*t* = 5 min, *T* = 6 °C, RPM = 10.000), and the supernatant was decanted using filter paper. The residue was extracted once more, and finally with hot water (75 °C, 20 mL) for 10 min. The resulting supernatants were pooled and vacuum-evaporated at 40 °C. The concentrated sugars were then redissolved in 20 mL of water and used for HPLC-ELSD analysis of soluble carbohydrates.

To determine the total amount of fructans in the obtained samples, 0.1 mL of inulinase solution was added (0.01% solution of Novozym 435) to a tube containing an aliquot of extracts of soluble carbohydrates (0.9 mL). The solution was mixed and incubated at 55 °C for 45 min, and the total released glucose and fructose was determined by HPLC.

Sugar content in all samples was determined by chromatographic analysis (HPLC-ELSD) following the method described by Nowicka et al. [43].

The concentration of fructans was calculated using the formula proposed by Hoebregs [44]:

Fructans = *k* × [(*G*_T_ − *S*/1.9 − (*G*_F_ + *G*_S/M_)) + (*F*_T_ − S/1.9 − *F*_F_) (2)

*G*_T_—total content of glucose

*S*—content of sucrose

*G*_F_—total content of free glucose

*G*_S/M_—total content of glucose from maltodextrin and starch

*F*_T_—total content of fructose

*F*_F_—total content of free fructose

All measurements were performed in triplicate and are presented as average values (g/100 g of product) ± SD.

### 3.5. Determination of Minerals by AAS

Fruit leathers were weighed into vessels at 1 g, with masses recorded in three decimal places. The samples were wet mineralized using 16 mL of a reagent composed of concentrated nitric acid (V) and concentrated hydrochloric acid in a 6:1 ratio, along with 1 mL of 30% hydrogen peroxide. Mineralization was performed using a digestion block system (Multiwave GO, Anton Paar, Graz, Austria), following the method described by Wojdyło et al. [45]. Mineral content was analyzed using an Atomic Absorption Spectrophotometer AA-7000 (Shimadzu, Kyoto, Japan) under standardized conditions. Measurements were performed in triplicate, and results are expressed as mg/100 g ± SD.

### 3.6. Determination of Polyphenols, Including Polymeric Proanthocyanidins by UPLC-PDA-FL

The extraction and determination of polyphenolic compounds in fruit leathers were performed according to Nowicka et al. [46]. Polyphenolic compounds were analyzed using an Acquity UPLC system (Waters Corp., Milford, MA, USA) equipped with a photodiode array and fluorescence detector. Retention times and absorbance values—280 nm for monomeric and dimeric flavan-3-ols, 360 nm for flavonols, 320 nm for phenolic acids, and 520 nm for anthocyanins—were compared with those of pure standards for compound identification. Additionally, the content of polymeric procyanidins was analyzed using the phloroglucinol method [47]. All measurements were performed in triplicate, and results are expressed as mg/100 g dry mass.

### 3.7. Analysis of Pro-Health Potential by In Vitro Methods

#### 3.7.1. Antioxidant Activity

The antioxidant properties of the samples were evaluated using three methods: ABTS, FRAP, and ORAC. The ABTS assay was conducted according to Re et al. [48], and the FRAP assay followed the method of Benzie et al. [49]. Both are colorimetric methods: in the ABTS assay, absorbance was measured at 734 nm, while in the FRAP assay, it was measured at 593 nm using a UV 2401 PC spectrophotometer (Shimadzu, Kyoto, Japan). The ORAC assay was conducted using the spectrofluorimetric method described by Ou et al. [50]. Measurements were taken at an excitation wavelength of 487 nm and an emission wavelength of 528 nm using an RF5301 PC spectrofluorometer (Shimadzu, Kyoto, Japan). Results for all methods are expressed as mmol of Trolox/100 g fresh sample ± SD, based on three repetitions.

#### 3.7.2. Ability to Inhibit α-Amylase and α-Glucosidase

The analysis was conducted using the colorimetric method described previously by Nowicka et al. [51], and a Synergy™ H1 microplate reader (BioTek, Winooski, VT, USA) was used for the measurements. For α-amylase inhibition, absorbance was measured at 540 nm, and for α-glucosidase inhibition, at 405 nm. Acarbose served as the positive control for both enzymes, with IC_50_ values of 0.35 and 0.20 mg/mL for α-amylase and α-glucosidase inhibition, respectively. The results are reported as IC_50_ ± SD (mg/mL).

### 3.8. Sensory Evaluation of Obtained Products

The sensory evaluation was conducted at the Faculty of Biotechnology and Food Sciences, Wrocław University of Environmental and Life Sciences, by ISO 8589:2009 standards [52]. The panel consisted of students specializing in Food Technology and Human Nutrition. They underwent comprehensive training, and ethical approval was obtained by national regulations (the Scientific Research Ethics Committee to conduct the sensory tests, as approved by resolution no. N0N00000.0020.1.8.2.2024). Participation was voluntary, and panelists were informed of this study’s objectives, with the option to withdraw at any time. Analyses of color, aroma, consistency, flavor, and overall desirability were performed using a 9-point hedonic scale, where 1 indicated dislike very much and 9 indicated like very much.

### 3.9. Statistical Analysis

All statistical analyses were performed using XLSTAT 2017 (Addinsoft, New York, NY, USA). The significance of differences (*p* ≤ 0.05) between means was evaluated using two-factor ANOVA followed by Duncan’s multiple range test.

## 4. Conclusions

The aim of this research was to develop innovative fruit leathers enriched with FOS derived from chicory and Jerusalem artichoke, known for their prebiotic properties and potential to influence the physicochemical, health-promoting, and sensory characteristics of the product. Peach, plum, red currant, and haskap berry were used in the formulations, mixed with pear puree. The products were prepared using the air-drying method, with FOS added at 10% of the mixture weight.

The incorporation of FOS reduced the total sugar content—particularly fructose and sorbitol—while increasing glucose or sucrose levels, depending on whether chicory or Jerusalem artichoke powder was used. The addition also lowered overall acidity and pectin content, due to the reduced proportion of fruit puree. At the same time, it significantly increased dietary fiber content, especially fructans, with the highest values observed in red currant and haskap berry leathers enriched with Jerusalem artichoke (up to 31.2%). FOS addition also altered the mineral profile, increasing levels of potassium, magnesium, calcium, and zinc—most notably in chicory-containing variants. Although the total polyphenol content decreased with FOS addition, certain fractions such as phenolic acids and flavonols increased due to the bioactive contributions of the FOS sources. Antioxidant activity was strongly influenced by the fruit matrix. Notably, fruit leathers containing chicory exhibited high α-glucosidase inhibitory potential. Formulations based on red currant, haskap berries, and peach with chicory achieved IC_50_ values comparable to acarbose, a commonly used antidiabetic drug. The inhibitory efficacy of Jerusalem artichoke was more dependent on the fruit type, suggesting that interactions between bioactive compounds in the fruit matrix and the FOS source play a significant role. The studies confirmed that enriching fruit leathers with FOS has a beneficial effect on their nutritional and health-promoting value. However, further sensory optimization, particularly in terms of taste and texture, is needed, with the bitterness of chicory remaining the most significant limiting factor. To improve sensory quality, it may be beneficial to apply pre-treatment methods, such as blanching or removing the chicory core, or to incorporate flavor maskers in the form of herbs, spices, or natural sweeteners. Modifying these sensory attributes will be crucial for achieving consumer acceptance and, consequently, for the market potential of the obtained products. The formulated products address several key needs of health-conscious consumers, offering high fiber content, low sugar levels, and a rich mineral profile. Their natural composition and clean-label character make them especially appealing to this target group. Additionally, the dried snack format offers practical benefits—it is lightweight, shelf-stable, easy to store and consume—making it well-suited for active, on-the-go lifestyles. Nevertheless, to fully realize their commercial potential, further work is needed to enhance sensory acceptability, extend shelf life, and assess the scalability of the production process for industrial application.

## Figures and Tables

**Table 1:** Physicochemical parameters of obtained fruit leathers.

Product	Color Parameters			Water Activity	Dry Matter	Energy Value	Titratable Acidity	pH
	L*	a*	b*		[%]	[kcal]	[g/100 g]	
1	22.31 ± 0.17 g	1.26 ± 0.13 h	−0.07 ± 0.04 g	0.327 ± 0.013 a	89.29 ± 0.07 c	337.52 ± 3.52 a	5.87 ± 0.04 d	3.141 j
2	22.66 ± 0.22 g	3.25 ± 1.73 g	0.30 ± 0.50 g	0.326 ± 0.012 a	88.04 ± 0.01 e	334.20 ± 5.13 a	4.91 ± 0.05 e	3.570 f
3	21.47 ± 0.61 g	1.67 ± 1.01 gh	−0.01 ± 0.27 g	0.326 ± 0.016 a	89.49 ± 0.14 bc	338.58 ± 5.47 a	5.06 ± 0.08 e	3.624 e
4	41.39 ± 1.27 b	43.66 ± 0.19 a	25.15 ± 0.21 b	0.314 ± 0.011 a	88.84 ± 0.06 d	327.84 ± 4.83 a	11.12 ± 0.07 a	2.805 k
5	35.12 ± 0.11 d	22.46 ± 0.71 c	14.42 ± 0.61 d	0.309 ± 0.009 a	88.51 ± 0.04 d	331.21 ± 5.44 a	6.78 ± 0.02 c	3.218 i
6	35.49 ± 0.92 d	27.38 ± 1.23 b	13.15 ± 0.51 de	0.314 ± 0.013 a	89.91 ± 0.34 a	334.44 ± 4.15 a	8.51 ± 0.04 b	3.247 h
7	52.16 ± 0.47 a	19.21 ± 0.60 d	49.10 ± 1.77 a	0.331 ± 0.004 a	88.11 ± 0.16 e	337.76 ± 5.40 a	3.75 ± 0.03 g	3.529 g
8	35.56 ± 1.60 d	12.70 ± 1.81 f	19.74 ± 2.60 c	0.328 ± 0.006 a	87.30 ± 0.20 f	333.87 ± 4.99 a	3.45 ± 0.04 hi	3.951 b
9	37.82 ± 2.18 c	15.39 ± 1.39 e	23.08 ± 3.38 b	0.329 ± 0.004 a	89.73 ± 0.16 ab	338.96 ± 5.37 a	3.28 ± 0.01 i	4.099 a
10	28.10 ± 1.19 f	22.56 ± 0.61 c	11.55 ± 0.77 e	0.321 ± 0.011 a	88.84 ± 0.01 d	335.85 ± 4.70 a	4.26 ± 0.01 f	3.251 h
11	31.83 ± 0.28 e	12.64 ± 1.16 f	11.34 ± 0.14 e	0.325 ± 0.008 a	87.79 ± 0.08 e	331.64 ± 5.78 a	3.61 ± 0.03 gh	3.712 d
12	30.24 ± 0.77 e	10.93 ± 0.49 f	8.96 ± 0.33 f	0.333 ± 0.006 a	87.81 ± 0.04 e	334.42 ± 4.53 a	3.77 ± 0.02 g	3.786 c

Data are given as mean ± standard deviation (*n* = 3). Mean values within a column with different letters (a–k) are significantly different (homogenous groups) at *p* ≤ 0.05.

**Table 2:** Different fractions of dietary fiber, and sugar content in obtained fruit leathers.

Product	Insoluble Dietary Fiber	Pectin—Soluble Dietary Fiber	Fructans—Soluble Dietary Fiber	Total Dietary Fiber	Fructose	Glucose	Sorbitol	Mannose	Sacharose	Total Sugar Content
	[g/100 g]									
1	14.93 ± 0.38 bc	8.33 ± 0.26 cd	0.19 ± 0.06	23.45 ± 0.70 d	20.61 ± 0.45a	2.28 ± 0.05 b	0.43 ± 0.01 d	nd	0.02 ± 0.00 i	23.34 ± 0.51 a
2	14.75 ± 0.34 c	5.28 ± 0.03 h	4.50 ± 0.18 b	24.53 ± 0.55 cd	14.44 ± 0.29 d	2.37 ±0.04 a	0.27 ± 0.01 gh	nd	0.03 ± 0.00 i	17.11 ± 0.34 e
3	15.77 ± 0.12 a	8.56 ± 0.04 c	6.89 ± 0.29 a	31.22 ± 0.45 a	13.89 ± 0.08 e	1.45 ± 0.05 f	0.28 ± 0.00 f	nd	0.13 ± 0.00 h	15.75 ± 0.13 f
4	14.58 ± 0.33 c	10.43 ± 0.24 a	0.00 ± 0.00 f	25.01 ± 0.57 c	17.13 ± 0.17 b	1.52 ± 0.05 e	0.30 ± 0.02 e	nd	nd	18.95 ± 0.24 b
5	14.62 ± 0.18 c	8.13 ± 0.20 de	1.42 ± 0.07 d	24.17 ± 0.45 cd	15.48 ± 0.25 c	2.39 ± 0.02 a	0.23 ± 0.00 i	0.03 ± 0.00 a	0.02 ± 0.00 i	18.15 ± 0.27 c
6	15.08 ± 0.22 b	8.62 ± 0.18 c	4.94 ± 0.16 b	28.64 ± 0.56 b	15.07 ± 0.30 c	1.13 ± 0.08 g	0.21 ± 0.01 i	nd	0.19 ± 0.02 g	16.60 ± 0.41 e
7	7.34 ± 0.05 h	10.68 ± 0.19 a	0.07 ± 0.00 e	18.09 ± 0.24 f	15.51 ± 0.09 c	1.07 ± 0.02 h	0.42 ± 0.00 d	nd	0.70 ± 0.01 b	17.69 ± 0.12 d
8	8.49 ± 0.01 f	6.54 ± 0.23 g	2.88 ± 0.29 c	17.91 ± 0.53 f	12.52 ± 0.11 f	1.98 ± 0.05 d	0.27 ± 0.00 gh	0.01 ± 0.00 b	0.38 ± 0.01 d	15.16 ± 0.17 g
9	8.03 ± 0.05 g	7.85 ± 0.23 e	7.36 ± 0.36 a	23.24 ± 0.64 d	12.70 ± 0.28 f	0.66 ± 0.07 i	0.26 ± 0.02 h	nd	0.72 ± 0.01 a	14.34 ± 0.38 h
10	11.29 ± 0.26 e	9.47 ± 0.07 b	0.00 ± 0.86 ef	20.76 ± 1.19 e	12.59 ± 0.03 f	1.53 ± 0.01 e	0.90 ± 0.02 a	nd	0.28 ± 0.00 e	15.30 ± 0.06 g
11	12.80 ± 0.17 d	6.78 ± 0.14 g	1.52 ± 0.13 d	21.11 ± 0.44 e	11.60 ± 0.18 g	2.15 ± 0.03 c	0.71 ± 0.02 c	0.01 ± 0.00 b	0.26 ± 0.01 f	14.73 ± 0.24 h
12	11.42 ± 0.08 e	7.40 ± 0.04 f	4.78 ± 0.33 b	23.60 ± 0.45 d	11.20 ± 0.09 g	1.19 ± 0.02 g	0.74 ± 0.01 b	nd	0.42 ± 0.01 c	13.55 ± 0.13 i

Data are given as mean ± standard deviation (*n* = 3). Mean values within a column with different letters (a–i) are significantly different (homogenous groups) at *p* ≤ 0.05; nd—not detected.

**Table 3:** Mineral content in different types of fruit leathers.

Product	Na	K	Mg	Ca	Fe	Zn	Mn
	[mg/100 g]						
1	3.02 ± 0.02 i	1360.09 ± 5.74 h	106.25 ± 0.67 k	41.24 ± 0.17 c	5.72 ± 0.04 ab	0.33 ± 0.00 g	0.45 ± 0.01 d
2	11.34 ± 0.08 c	1893.10 ± 12.17 e	213.55 ± 0.83 d	42.00 ± 25 c	5.69 ± 0.05 ab	0.68 ± 0.01 b	0.61 ± 0.00 a
3	3.18 ± 0.05 i	1924.27 ± 15.85 e	141.31 ± 0.55 i	27.06 ± 0.26 f	5.48 ± 0.04 ab	0.46 ± 0.00 e	0.37 ± 0.00 e
4	7.92 ± 0.11 e	1519.59 ± 4.05 g	124.53 ± 0.24 j	43.34 ± 0.35 b	6.27 ± 0.04 a	0.31 ± 0.01 g	0.29 ± 0.00 f
5	12.18 ± 0.09 a	2650.89 ± 17.22 b	246.02 ± 1.11 b	44.96 ± 0.24 a	6.26 ± 0.04 ab	0.69 ± 0.00 b	0.61 ± 0.00 a
6	4.82 ± 0.05 f	2874.02 ± 22.80 a	154.36 ± 2.81 g	35.38 ± 0.34 d	6.34 ± 0.09 a	0.43 ± 0.02 e	0.29 ± 0.00 f
7	3.23 ± 0.01 i	1776.41 ± 21.91 f	160.26 ± 0.65 f	20.18 ± 0.30 h	5.77 ± 0.04 ab	0.56 ± 0.00 d	0.31 ± 0.00 f
8	11.68 ± 0.01 b	2616.19 ± 16.05 b	310.96 ± 4.23 a	29.06 ± 0.54 e	6.18 ± 0.06 ab	0.91 ± 0.02 a	0.58 ± 0.00 b
9	3.56 ± 0.08 h	2419.21 ± 23.13 c	169.04 ± 0.88 e	17.98 ± 0.41 i	5.92 ± 0.07 ab	0.63 ± 0.03 c	0.26 ± 0.00 g
10	2.78 ± 0.04 j	1291.56 ± 20.00 h	121.58 ± 0.27 j	11.77 ± 0.08 k	5.28 ± 0.03 b	0.23 ± 0.01 h	0.31 ± 0.00 f
11	10.92 ± 0.11 d	2154.35 ± 8.52 d	230.88 ± 1.07 c	21.34 ± 0.33 g	5.94 ± 0.04 ab	0.57 ± 0.00 d	0.52 ± 0.00 c
12	4.47 ± 0.07 g	1959.86 ± 9.16 e	148.96 ± 2.58 h	13.67 ± 0.19 j	5.660.07 ab	0.37 ± 0.00 f	0.30 ± 0.00 f

Data are given as mean ± standard deviation (*n* = 3). Mean values within a column with different letters (a–k) are significantly different (homogenous groups) at *p* ≤ 0.05.

**Table 4:** Antioxidant activity, and inhibition effect against α-amylase and α-glucosidase of obtained fruit leathers.

Product	ABTS	FRAP	ORAC	α-Amylase	α-Glucosidase
	[mmol Trolox/100 g]			IC_50_ [mg/mL]	
1	9.91 ± 0.68 a	7.20 ± 0.09 b	18.88 ± 2.72 b	84.20^0.9424^ j	2.84^0.9515^ h
2	7.36 ± 1.13 bc	5.97 ± 0.01 c	18.76 ± 0.82 b	86.18^1.0000^ j	<0.50^0.9677^ i
3	6.94 ± 0.10 c	5.78 ± 0.17 c	19.56 ± 0.77 a	100.20^0.9999^ i	15.05^0.8674^ g
4	5.74 ± 0.65 d	4.90 ± 0.03 e	10.39 ± 0.99 d	14.71^0.8423^ k	18.81^0.9959^ f
5	2.32 ± 1.01 g	2.21 ± 0.39 g	11.10 ± 1.33 c	131.29^0.8669^ f	<0.50^0.9527^ i
6	2.65 ± 0.15 g	2.20 ± 0.04 g	9.11 ± 0.19 e	112.33^0.8485^ h	23.79^0.9510^ e
7	4.32 ± 0.14 e	3.40 ± 0.02 f	7.21 ± 0.33 g	208.62^0.8989^ d	27.60^0.9999^ d
8	2.59 ± 0.12 g	2.13 ± 0.03 g	6.27 ± 0.38 h	231.93^0.9913^ b	<0.50^0.9981^ i
9	3.26 ± 0.16 f	2.34 ± 0.02 g	5.91 ± 0.42 h	224.51^0.9842^ c	80.08^0.9976^ a
10	9.98 ± 0.10 a	7.60 ± 0.24 a	9.06 ± 0.27 e	199.50^0.9984^ e	26.68^0.9711^ d
11	7.74 ± 1.05 b	5.51 ± 0.13 d	6.06 ± 0.09 h	121.06^0.9973^ g	56.78^0.9988^ b
12	7.02 ± 0.30 c	5.15 ± 0.10 e	7.95 ± 0.61 f	370.15^0.8866^ a	55.17^0.9620^ c

Data are given as mean ± standard deviation (*n* = 3) (antioxidant activity). IC_50_ values were determined based on 6 repetitions for 3 concentrations of compounds—the superscript is the R coefficient. Mean values within a column with different letters (a–k) are significantly different (homogenous groups) at *p* ≤ 0.05.

## Data Availability

All related data and methods are presented in this paper. Additional inquiries should be addressed to the corresponding authors.

## Supplementary Materials

The following supporting information can be downloaded at: https://www.mdpi.com/article/10.3390/molecules30122507/s1, Table S1: Content of anthocyanins, flavonols, phenolic acids, flavan-3-ols, and polymeric proanthocyanidins in obtained fruit leathers; Table S2: Physical properties, dietary fiber, and sugar content in raw materials used to fruit leathers production; Table S3: Polyphenolic compounds in raw materials used to fruit leathers production.

## Abbreviations

The following abbreviations are used in this manuscript:

FRAP	Ferric Reducing Ability of Plasma
ORAC	Oxygen Radical Absorbance Capacity
FOS	fructooligosaccharides
