# Supplementary material for: Functional Fruit Snacks Enriched with Natural Sources of Fructooligosaccharides: Composition, Bioactive Compounds, Biological Activity, and Consumer Acceptance
Source: Molecules. 2025 Jun 7;30(12):2507. doi: 10.3390/molecules30122507 (PMC12195991; doi:10.3390/molecules30122507)
Supplement: Supplementary file 1 [file molecules-30-02507-s001.zip › molecules-3676587-supplementary.pdf]

Table S1. Content of anthocyanins, flavonols, phenolic acids, flavan-3-ols, and polymeric proanthocyanidins in obtained fruit leathers

Product	Anthocyanins	Flavonols	Phenolic acids	Flavan-3-ols (monomers & dimers)	Polymeric proanthocyanidins	Total polyphenols
**1**	240.16±6.37 a	72.53±1.05 c	90.98±2.78 c	61.20±0.53 c	239.36±0.85 a	704.23±11.58 a
**2**	203.98±2.96 c	121.03±1.05 a	139.71±4.26 a	65.38±0.48 b	144.22±0.43 d	674.34±9.18 b
**3**	224.84±3.22 b	99.43±0.74 b	103.53±3.48 b	33.57±0.69 e	197.76±0.62 b	659.14±8.74 b
**4**	10.03±0.10 e	6.25±0.05 f	15.40±0.08 h	108.07±0.94 a	152.45±0.56 c	292.21±1.73 c
**5**	2.42±0.02 f	5.60±0.14 g	36.18±0.51 f	35.90±0.91 d	75.51±1.73 i	155.62±2.82 e
**6**	3.45±0.05 d	3.27±0.05 j	18.85±0.50 g	13.39±0.52 f	92.87±1.57 h	131.84±2.70f
**7**	nd	nd	10.97±0.30 i	4.32±0.33 i	112.46±1.54 f	127.76±2.34 f
**8**	nd	3.54±0.05 i	36.19±0.19 f	8.56±0.29 h	41.14±0.09 j	89.44±0.62 g
**9**	nd	5.07±0.47 h	36.82±1.37 ef	9.44±0.55 g	71.79±4.18 i	123.11±6.17 f
**10**	1.93±0.02 h	6.95±0.07 e	39.78±0.27 e	4.09±0.06 i	127.37±0.46 e	180.13±0.88 d
**11**	2.36±0.06 f	14.44±0.23 d	58.50±0.94 d	8.71±0.15 h	77.32±1.73 i	161.34±3.30 e
**12**	2.16±0.04 g	4.70±0.27 k	38.90±0.67 e	10.01±0.57 g	106.24±1.32 g	161.99±2.86 e

nd – not detected; data are given as mean ± standard deviation (*n* = 3). Mean values within a column with different letters (a–k) are significantly different (homogenous groups) at $p \leq 0.05$.

Table S2. Physical properties, dietary fiber, and sugar content in raw materials used to fruit leathers production

**Raw material**	**Colour parametrs**			**Water activity**	**Dry matter**	**Insoluble dietary fiber**	**Pectin - soluble dietary fiber**	**Fructans - soluble dietary fiber**	**Total dietary fiber**	**Fructose**	**Sorbitol**	**Glucose**	**Sacharose**	**Total sugar content**
	**L***	**a***	**b***		**[%]**	**[g/100 g dm]**								
Jerusalem artichoke	54.44±0.05	0.44±0.01	10.93±0.02	0.247±0.009	94.09±0.03	17.23±0.13	1.49±0.03	57.30±2.13	76.02±2.29	1.71±0.33	nd	1.74±0.05	6.52±0.01	9.97±0.39
Chicory	40.77±0.03	7.12±0.01	24.26±0.01	0.264±0.021	95.17±0.07	19.78±0.47	0.04±0.01	23.85±0.70	43.67±1.18	2.13±0.07	nd	6.09±0.40	1.32±0.02	9.54±0.49
Pear	-	-	-	-	16.13±0.02	8.68±0.15	4.59±0.03	nd	13.27±0.18	49.54±1.07	11.41±0.03	8.99±0.06	nd	69.94±1.16
Haskap berry	-	-	-	-	15.67±0.00	9.57±0.17	3.83±0.02	nd	13.40±0.19	24.53±0.40	2.15±0.03	22.23±0.65	nd	48.91±1.08
Red currant	-	-	-	-	14.52±0.02	15.15±0.37	8.26±0.11	nd	23.41±0.48	18.07±0.05	nd	13.85±0.27	nd	31.92±0.32
Peach	-	-	-	-	11.21±0.08	9.81±0.11	9.63±0.36	nd	19.44±0.47	7.76±0.10	nd	7.26±0.21	32.73±0.56	47.75±0.87
Plum	-	-	-	-	16.35±0.00	7.34±0.04	8.01±0.12	nd	15.35±0.16	10.36±0.23	6.57±0.00	15.25±0.07	5.31±0.07	37.49±0.37

dm – dry matter; nd – not detected; data are given as mean ± standard deviation (*n* = 3)

Table S3. Polyphenolic compounds in raw materials used to fruit leathers production

**Raw material**	**Anthocyanins**	**Flavonols**	**Phenolic acids**	**Flavan-3-ols (monomers & dimers)**	**Polymeric proanthocyanidins**	**Total polyphenols**
	**mg/100 g dm**					
Jerusalem artichoke	nd	4.19±0.06	169.28±4.98	nd	106.62±1.23	280.09±6.27
Chicory	nd	75.27±1.14	465.75±7.70	16.75±0.08	53.48±0.72	611.25±9.64
Pear	nd	21.13±0.07	169.98±5.01	81.59±2.67	258.36±4.01	531.06±11.76
Haskap berry	2044.58±31.59	134.16±1.84	269.18±5.72	162.49±2.99	2215.46±7.73	4825.87±49.87
Red currant	225.51±5.21	32.16±1.99	15.09±0.72	155.88±5.12	962.52±11.52	1391.16±24.56
Peach	55.60±0.05	13.24±0.71	207.09±3.87	20.18±0.23	892.27±13.77	1188,38±18.63
Plum	nd	17.32±0.05	221.59±6.02	65.69±1.00	902.97±7.31	1207.57±14.38

dm – dry matter; nd – not detected; data are given as mean ± standard deviation (*n* = 3)
